# Points in Medical Ethics

**Published:** 1888-08

**Authors:** J. E. Mayfield

**Affiliations:** Nacogdoches, Texas


### POINTS IN MEDICAL ETHICS.

By J. E. Mayfield, M. D., Nacogdoches, Texas.

For Daniel's Texas Medical Journal.

THE term ethics properly applies to, and involves questions of right and wrong, especially as to professional subjects. The relations of the medical profession and the public—the duties of each to the other—are generally misunderstood by the public, and even by persons who would be thought more intelligent. It is truly surprising, to find that all explanation fails to reach them to the extent of enabling them to fairly comprehend the principles and ethics of the profession of medicine.

The true reason of this blindness is that the general public is deluded by viewing these questions, as they do most others, from what is considered a "business" standpoint, or, more properly, a mercenary or money-seeking point of view. Physicians are regarded as being a class of business men, in the ordinary sense, following their profession exclusively for gain to themselves, and they are expected to resort to the usual means and methods in the scramble to attain this end. The term "success," in its business sense, means gain to its worker, without reference to doing good to others. Physicians are thought to desire this kind of success.

The blacksmith, the house painter, the brickmason, and the laborer in most other occupations, will seek advantages, bid against his neighbor for jobs, and scheme for profits. The merchant considers it fair to win customers from his brother merchants, to make profits both in buying and selling, and to turn various plans for success in business, and he who has the hardest heart, as a rule, has the greatest success. And so the lawyer, the doctor, and even the preacher is expected to do likewise. Schemes and tricks in this direction are expected of them. The general idea is, every fellow for himself, and the devil take the hindmost.

Now, there is nothing more mortifying and humiliating to an honorable physician than to be rated in this sense. It is admitted that one expects to make a living, and sometimes more, by his labors, and that he requires reasonable compensation for his services. It is admitted that there are black sheep in the profession, plying their arts for greedy gain ; that charlatans, quacks and imposters are abroad, under the guise of physicians ; and al physicians deplore these facts.

But the regular medical profession is laboring as well for the public good and for individual clients, as for self-interest. They search the depth of science ; explore the fields of knowledge ; labor hard at burdensome tasks, and give to the world the benefit of every attainment. The courts, the legislatures, the country, all are recipients of their gifts, and are guided by their knowledge. They are not only willing servants of the public, but are often heroes in the public defense. Not only do they warn the public of dangers and suggest the means of escape, but often freely face the danger and battle with the enemy—be it small-pox, yellow fever, cholera, or war's bloody experiences. Little does the average citizen think of the trials that the doctor undergoes, the sacrifices of self-interest that he makes for the public welfare.

So, when the medical profession petitions the legislature to enact laws to regulate the practice of medicine, desiring to protect the people from the impositions and frauds of medical humbugs, quacks and incompetent practitioners, the public fails to see that their motives are pure, but suspects that they are actuated by self-interest and hope to reduce the number of doctors, and thus get a monopoly of the "business." If the profession condemn quacks, patent medicines, or medical humbugs, it is suspected that they are jealous of them and desire their suppression to increase their own gain. What greater mistake could be made?

The Galveston News, a paper that is watchful of the public interest, fearless in advocating its principles, and unsurpassed in its intellectual make up, is to be numbered with the blind on some of the leading principles of medical ethics. In the daily of May 8, 1888, it published the address of the committee of the Texas State Medical Association to the people, in which is shown the importance and necessity of laws regulating the practice of medicine, under a heading containing the words "Protesting Physicians. * * * Some good reasons why the State should grant them the desired protection," and concludes its brief and disparaging editorial notice of the address thus: "But if the State were to undertake medicine in earnest, it might logically go to the extent of providing public physicians, with adequate pay, for the general treatment of the sick. * * * The theory of the Texas Medical Association is highly paternal. Its development will form an interesting subject of intelligent scrutiny."

The same paper, June 1, 1888, has an editorial headed, "Need of a higher professional standard," in which it strongly advocates the purification of the profession of law, and the elevation of the bar "to a degree that would eliminate the whole tribe of barratry, shystering and black-mailing sharps who ply their trade in the imposing guise of lawyers," and incidentally refers to the Texas State Medical Association as being "anxious to rid their profession of speculative charlatans and unscrupulous adventurers." Proceeding further, it makes a comparison of the two professions, giving advantages to the law, and says that "physicians have no official status, as do the lawyers." The inference is, that the public purse, as affected by the profession of law, takes rightful precedence over the public health, and the purse thrown in, as affected by the medical profession. Let impostors in medicine kill and steal, but let not such be done in law. Protect the public on the one hand, where only the purse is involved, but on the other, where purse and health, and even life itself, is involved, let the public look out for itself. How can people fail to see that there is more evil to them in a villainous doctor or humbug medicine vendor, than in a villainous lawyer, or teacher, or preacher, or what not? An incompetent lawyer or teacher is not to be compared with an incompetent physician; neither is dishonesty in the one to be compared with that of the other. The physician is intrusted with the health, the life, the family honor, with all that is dear to his *clientele;* wife, daughter and child, health, honor, family secrets, everything involving the question of human happiness, is in his care. And what if he be a villain, or even incompetent for his high responsibility and sacred duty? What greater need can there be than for public protection against such evils?

Yet, the public refuses to listen to the warnings of its most trusted servants—the medical profession. We are not egotistical, immodest nor selfish, when we respond to the call of duty in claiming to know what is best for the people in our line, or claiming that we are entitled to credence in our warnings as to the dangers that surround the public. We feel it our conscientious duty to advise the public in these matters, and to persuade and help them to take steps for their protection.

The legislature should be required to pass laws regulating the practice of medicine, and its kindred subjects, whereby humbugs, frauds and villiany may be prevented, such as are well known to exist; and to prevent epidemics, keep out and suppress contagious diseases, and, in general, to superintend and care for the public health. The medical profession should be looked to for guidance in all such matters. If they are worthy of ordinary professional confidence, surely they can be entrusted with questions of public hygiene.

